# No Difference in Keratin Thickness between Inner and Outer Foreskins from Elective Male Circumcisions in Rakai, Uganda

**DOI:** 10.1371/journal.pone.0041271

**Published:** 2012-07-18

**Authors:** Minh H. Dinh, Taha Hirbod, Godfrey Kigozi, Eneniziaogochukwu A. Okocha, Gianguido C. Cianci, Xiangrong Kong, Jessica L. Prodger, Kristina Broliden, Rupert Kaul, David Serwadda, Maria J. Wawer, Ronald H. Gray, Thomas J. Hope

**Affiliations:** 1 Departments of Infectious Diseases and Cell and Molecular Biology, Northwestern University Feinberg School of Medicine, Chicago, Illinois, United States of America; 2 Karolinska Institutet, Department of Medicine Solna, Center for Molecular Medicine, Stockholm, Sweden; 3 Rakai Health Sciences Program, Entebbe, Uganda; 4 Department of Medicine, University of Toronto, Toronto, Ontario, Canada; 5 School of Public Health, Makerere University, Kampala and Rakai, Uganda; 6 Bloomberg School of Public Health, Johns Hopkins University, Baltimore, Maryland, United States of America; Asociacion Civil Impacta Salud y Educacion, Peru

## Abstract

It has been hypothesized that increased HIV acquisition in uncircumcised men may relate to a more thinly keratinized inner foreskin. However, published data are contradictory and potentially confounded by medical indications for circumcision. We tested the hypothesis that the inner foreskin was more thinly keratinized than the outer foreskin using tissues from 19 healthy, HIV-uninfected men undergoing routine prophylactic circumcision in Rakai, Uganda. Sections from 3 foreskin anatomic sites (inner, outer, and frenar band) were snap-frozen separately. Two independent laboratories each separately stained, imaged, and measured keratin thicknesses in a blinded fashion. There was no significant difference in keratin thickness between the inner (mean = 14.67±7.48 µm) and outer (mean = 13.30±8.49 µm) foreskin, or between the inner foreskin and the frenar band (mean = 16.91±12.42 µm). While the frenar band showed the greatest intra-individual heterogeneity in keratin thickness, there was substantial inter-individual variation seen in all regions. Measurements made by the two laboratories showed high correlation (r = 0.741, 95% CI, 0.533–0.864). We conclude that, despite inter- and intra-individual variability, keratin thickness was similar in the inner and outer foreskin of healthy Ugandan men, and that reduced keratin thickness is not likely to make the inner foreskin more susceptible to HIV acquisition.

## Introduction

Three prospective randomized clinical trials conducted in Africa have shown that male circumcision protects against HIV acquisition [1], [2], [3]. In addition, male circumcision has been shown to significantly reduce rates of human papillomavirus (HPV) and herpes simplex virus-2 (HSV) [4], [5]. It has been hypothesized that HIV acquisition in an intact foreskin occurs primarily across the inner foreskin surface, due to a putatively thinner keratin layer in this region [6], [7], [8]. This would allow for easier HIV entry across the epithelium, with subsequent access to a pro-inflammatory immune milieu that is enriched for highly HIV-susceptible CD4+ T cell subsets [9], [10]. In contrast, a thicker stratum corneum (cornified or keratin layer) in the outer foreskin and penis has been hypothesized to provide a more robust barrier to viral entry [11]. A few groups have examined this theory: three studies supported this notion and three found either no difference or a thicker inner foreskin keratin layer [6], [7], [12], [13], [14], [15].

One of the latter studies from our group demonstrated no reduced inner foreskin keratin thickness using de-identified samples obtained from volunteers in Chicago, IL, USA [15]. However, the inclusion of donors with balanitis (foreskin inflammation) or phimosis (difficulty retracting the foreskin) was critiqued, as these clinical indications for circumcision may have confounded our results [16], [17]. Additionally, donor race or ethnicity may have affected our results and hence generalizability to the men of African ancestry who were enrolled in the aforementioned male circumcision trials.

The current study addresses these concerns by utilizing specimens from healthy African men. Foreskin tissues were collected from donors in Rakai, Uganda receiving circumcision as a prophylactic measure against HIV and sexually transmitted infections (STI). Two independent laboratories then analyzed samples of inner, outer, and frenar band foreskin (the latter region lies between the inner and outer foreskin, comes into close contact with the glans penis, and may be more susceptible to HIV entry) [16]. This multi-site collaborative effort examined the existence of thinly keratinized foreskin keratin layers, which might contribute to greater HIV susceptibility in uncircumcised men.

## Methods

### Sample Collection

These studies were approved by institutional review boards at the Uganda Virus Research Institute, the Uganda National Council of Science and Technology, the University of Toronto, the Regional Ethical Review Board in Stockholm, Sweden, Johns Hopkins University, and Northwestern University. Adult males in Rakai, Uganda are currently offered free male circumcision as a prophylactic measure against HIV and other STIs. Study participants provided written informed consent for the collection and analysis of normally discarded foreskin tissues. Interviews and physical exams were conducted to ascertain concurrent medical conditions such as evidence of pre-existing STIs or ulcers and any such conditions were treated prior to surgery. Circumcision was performed using the dorsal slit method, and the inner foreskin was marked during the procedure. Foreskin samples were then immediately separated into three anatomic sites: inner, outer, and frenar band (JP, RK), using methods previously described [15]. The frenar band was identified as the transition region between inner and outer foreskin. Small sections from each site were snap-frozen in plastic cryomolds containing OCT (optimal cutting temperature, Sakura Finetek, Torrance, CA) and labeled with corresponding letters, ‘A,’ ‘B,’ or ‘C.’ These sections were then shipped frozen to the Karolinska Institutet (Stockholm, Sweden) for blinded preparation and analysis (TH).

**Table 1:** Demographic and epidemiologic data of male participants.

	*n* = 19
Mean age (years)	29.2 (range = 21–41)
Marital status	
Single	4 (21%)
Married	14 (74%)
Polygamous marriage	2 (11%)
Not specified	1 (5%)
Sexually Active	
Current	17 (89%)
Not anymore	2 (11%)
Never	1 (5%)
Sexual Partners (last 12 months)	
1	11 (58%)
2	3 (16%)
3 or more	3 (16%)
No Answer	2 (11%)
Condom Use	
Never	10 (53%)
Sometimes	3 (16%)
Always	5 (26%)
Not applicable	1 (5%)
History of symptoms consistent with STIs	
No	19 (100%)

**Figure 1:**
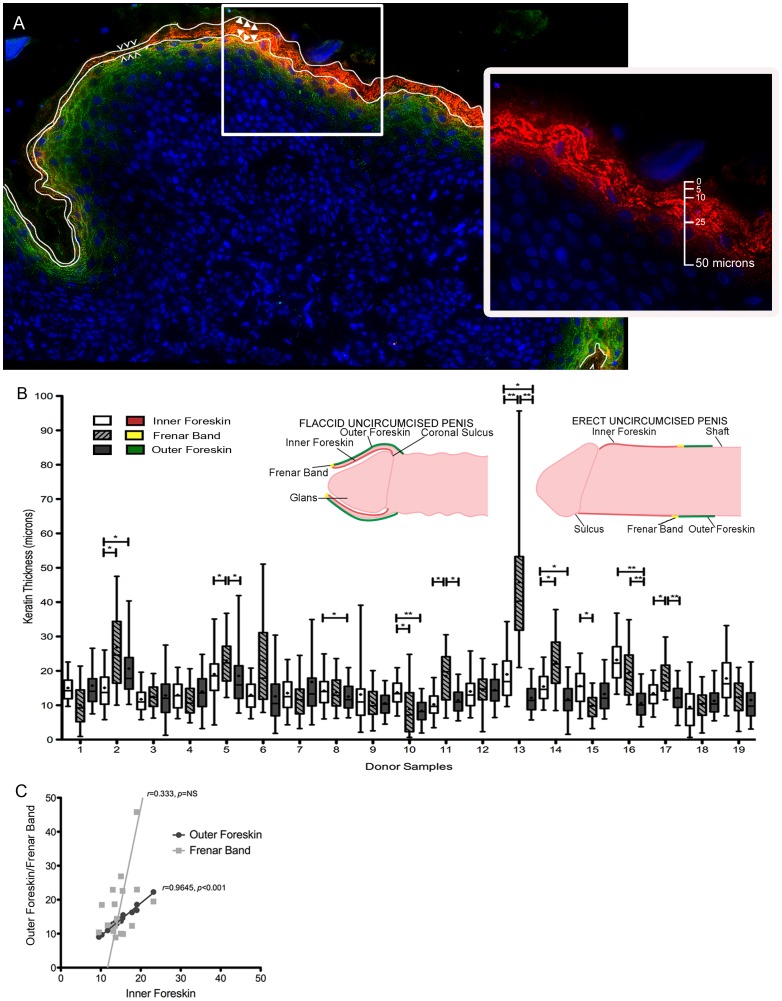
Mean foreskin keratin thicknesses by donor and region. (A) Representative image of intra-individual variation in keratin thickness (outer foreskin). White ‘**⁁⁁⁁**’ symbols show areas of thinner keratin and white solid triangles show areas of thicker keratin. Involucrin (green), filaggrin (red), cell nuclei (blue). Inset: magnification of boxed area with only filaggrin staining (red) shown; scale depicts thickness in microns. (B) Illustration of foreskin regions examined: inner (red), frenar band (yellow), and outer (green) foreskin. Whisker and boxplots of individual keratin measurements demonstrating distribution of thicknesses. Median  =  middle line, box = 25–75^th^ percentiles, whiskers = 5–95^th^ percentiles, mean  =  ‘+’ symbols, outliers not shown for ease of viewing. *p<0.05, **p<0.001. (C) Correlation of mean regional measurement per donor. Each symbol represents mean thickness calculated for one donor. Light gray boxes/line = inner: frenar band ratio and dark gray circles/line = inner: outer ratios.

**Table 2:** Individual keratin thickness measurements by anatomic foreskin region.

	Inner Foreskin	Outer Foreskin	Frenar Band	*Inner:Outer*	*Outer:Frenar*	*Inner:Frenar*
Donor Number	Mean ± SD*	Mean ± SD	Mean ± SD	*p*-value**		
1	15.02±4.34	15.72±9.32	10.02±6.27	0.633	0.104	0.133
2	15.05±7.91	20.66±9.35	26.87±12.86	**0.044**	0.077	**0.012**
3	11.73±5.43	12.88±8.41	12.47±4.14	0.338	0.773	0.433
4	12.98±4.72	14.00±7.08	11.94±6.52	0.739	0.425	0.381
5	19.04±10.17	18.56±10.86	22.98±7.97	0.710	**0.040**	**0.050**
6	13.01±4.65	12.60±8.38	22.94±14.21	0.928	0.101	0.101
7	13.56±7.04	16.80±16.78	12.32±4.02	0.340	0.070	0.512
8	14.19±5.31	12.54±4.81	13.75±5.24	**0.002**	0.346	0.831
9	13.14±10.73	10.58±3.95	10.75±5.48	0.207	0.709	0.331
10	13.71±4.16	8.37±3.98	8.90±7.67	**0.001**	0.820	**0.010**
11	10.27±4.27	11.44±4.23	18.48±8.15	0.375	**0.030**	**0.017**
12	14.02±6.05	14.39±4.66	14.38±5.75	0.997	0.502	0.559
13	18.99±8.26	12.14±5.01	45.79±22.00	**0.050**	**0.000**	**0.000**
14	15.46±5.39	11.74±6.29	22.61±9.06	**0.031**	0.158	**0.031**
15	15.60±6.13	13.29±5.95	9.90±4.99	0.081	0.058	**0.008**
16	23.19±7.18	10.62±4.77	19.47±7.85	**0.000**	**0.001**	0.074
17	13.47±4.80	12.18±4.55	18.66±7.30	0.146	**0.001**	**0.004**
18	9.52±6.69	11.27±4.71	10.39±4.54	0.298	0.545	0.231
19	17.81±7.76	11.53±8.98	12.29±5.52	0.300	0.380	0.093

* SD =  standard deviation.

** *p*-value based on two-tailed Student’s t-test.

Bolded *p*-values indicate significantly thinner inner, outer, or frenar band regions.

### Immunohistochemistry

Thin (∼10 µm thickness) cryosections were obtained from each sample and placed onto glass slides. Tissues were fixed in 0.1M PIPES buffer, pH 6.8 and 3.7% formaldehyde (Polysciences). One set of frozen, blinded slides was kept at the Karolinska Institutet, and a second was shipped to Northwestern University (Chicago, IL, USA) for analysis (EO, MD). Upon arrival at Northwestern University, slides were prepared as previously described and the keratin layer was highlighted with fluorescent markers for filaggrin and involucrin [18]. A concurrent, separate analysis was conducted at the Karolinska Institutet using the same methods (TH, KB).

**Figure 2:**
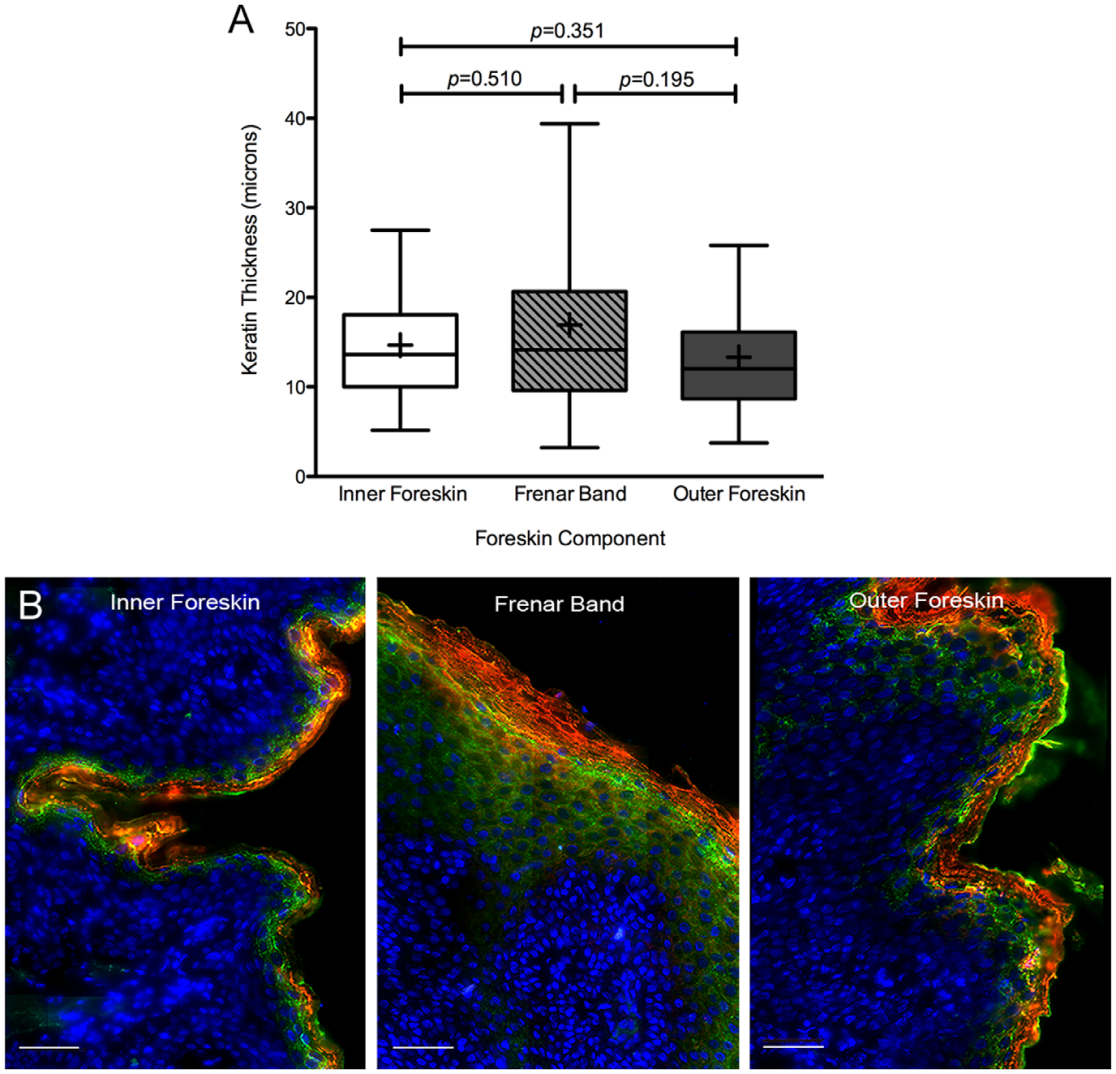
Cumulative foreskin keratin thicknesses by region. (A) Whisker and boxplots of cumulative keratin thicknesses per region: inner foreskin (white), frenar band (light gray), outer foreskin (dark gray). Median  =  middle line, mean  =  ‘+’ symbol, outliers not shown for ease of viewing. (B) Representative tissue from each region stained for filaggrin (red), involucrin (green), and cell nuclei (blue). White scale bars = 50 µm.

### Imaging and Analysis

At Northwestern, imaging was conducted using DeltaVision RT systems and SoftWorx software (Applied Precision Instruments, API, Issaquah, WA). For each tissue section, three sequential images (panels) were captured at 60x magnification and stitched panels were quick projected for analysis. An interactive program was written (GC) using the programming language IDL (Interactive Data Language, ITTVIS Boulder, CO). The program functions as follows: using a tablet and stylus system, the user marks the boundaries of interest on the immunostained image of A) the epithelial surface, and B) basal edge of the keratin (figure S1A). The software then measures the closest distance from A to B for each point along A. In order to account for errors close to the image edge or those due to significant tissue curvature, the process is repeated in the opposite direction (B to A) (figure S1B). For each of the three foreskin regions, three sequential fields of view were measured. Approximately 10,000 points per donor region were used to calculate keratin thickness.

**Figure 3:**
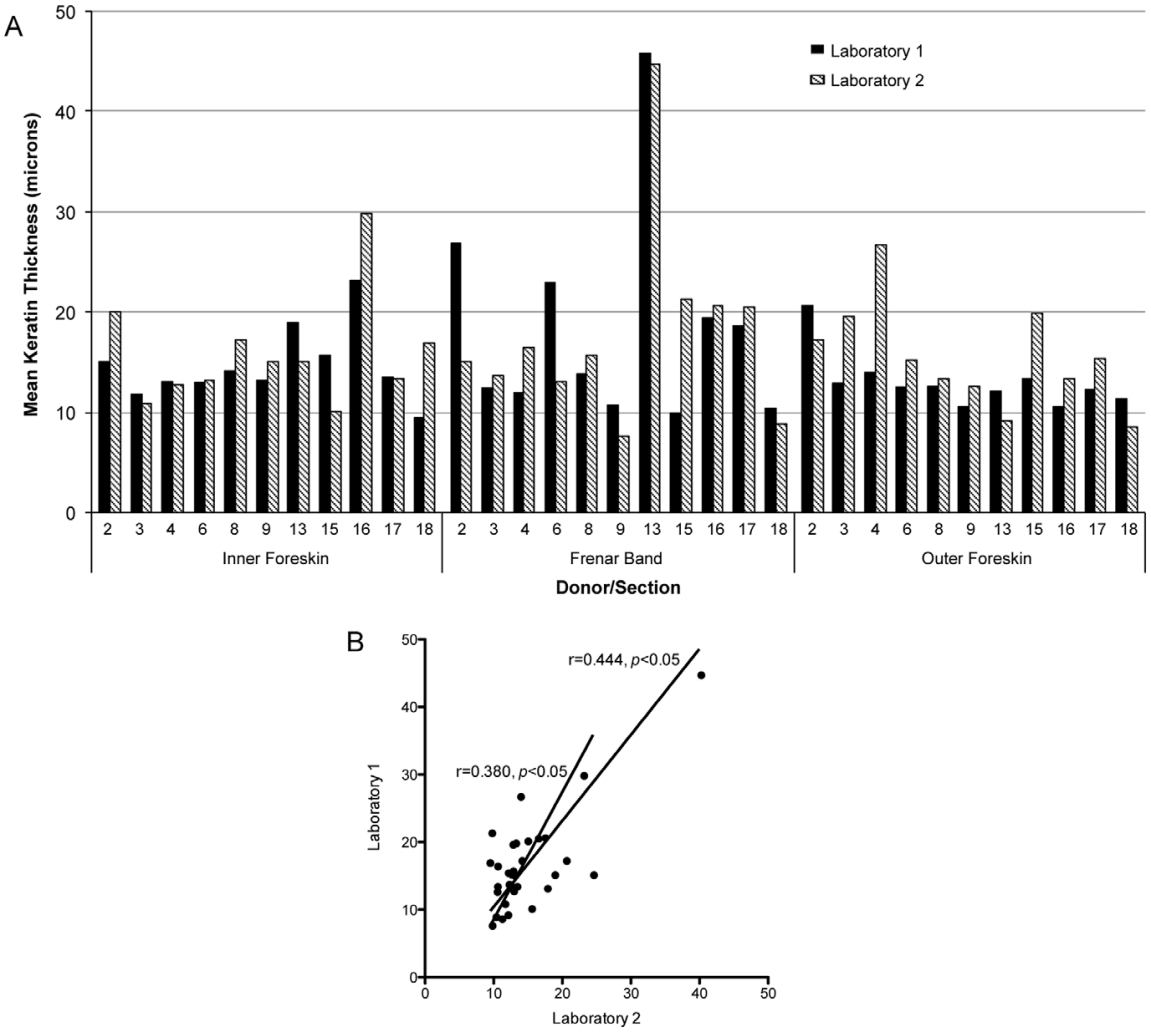
Data obtained from two independent laboratories. (A) Mean thicknesses recorded by laboratory 1 (black bars) and laboratory 2 (hatched bars) for 11 donors. (B) Spearman rank correlation of two laboratory measurements. Shorter line  =  correlation coefficient calculated excluding donor sample #13.

Imaging at the Karolinska Institutet was conducted with Qwin 550 software and a filter-free spectral confocal microscope (Leica TCS SP2 AOBS). Images were captured at 250x magnification, representing approximately 1500 µm of the epithelial surface for each donor sample and foreskin anatomic site. Keratin thickness was measured as previously described with a few alterations [10], [15]: images were analyzed using Adobe Photoshop CS5 and sequential measurements were repeated every 40 µm along the epithelial surface (Adobe Systems Incorporated, San Jose, CA). This manual method was significantly more time-consuming. Therefore, the accumulated data from the Karolinska Institutet at the time in which the Northwestern analysis was completed was used for comparison purposes.

### Statistical Analysis

For each donor, paired, two-tailed Student’s t-tests and Spearman rank correlation coefficients were used to evaluate inner:outer, inner:frenar band, and outer:frenar band mean keratin thicknesses (Prism 5, GraphPad, La Jolla, CA). Linear mixed effect model was used to compare mean overall measurements among the three anatomic foreskin sites (XK, SAS 9.22). Specifically, the anatomic site was modeled as a fixed effect, and the unstructured residual covariance matrix model was chosen to examine the residual variance-covariance matrix (based on the restricted maximum likelihood method and Kenward-Roger approach of calculating the denominator degrees of freedom for parameter estimation and hypothesis testing, respectively [19]). Model diagnostics were conducted to assess normality assumption and influential data points. The Sidak p-value adjustment method was used for multiple comparisons of each site. All tests were two-sided with significance level of 0.05. Finally, Spearman rank correlation coefficients were used to evaluate the two laboratory analyses (Prism 5, GraphPad, La Jolla, CA).

## Results

Foreskin samples from 19 HIV-seronegative adult male donors were included in our analysis. Donors’ baseline demographic information is shown in table 1. The average age of the donors was 29.2 years. The majority of the subjects were married. Approximately half reported monogamous sexual relationships, which was fewer than those who reported marriage. Most participants were sexually active, and only 26% reported consistent condom use. No participants reported urethral discharge, genital warts or ulcers, or pain with foreskin retraction prior to surgery. Physical exam at the time of surgery revealed that one donor (#18) had very mild foreskin inflammation, or balanitis. He was treated and returned 2 weeks later for circumcision when the inflammation had resolved. Another asymptomatic donor had hypospadias on exam (#9). Neither factor correlated with observable differences in foreskin keratin thickness.

We observed substantial heterogeneity in keratin thickness within each anatomic foreskin site for every donor (figures 1A and 1B and table 2). Within each donor, the mean keratin thickness of each anatomic site as compared to other sites also varied substantially (figure 1B). In 7 of 19 donor samples, mean frenar band keratinization was significantly thicker than that of inner, outer, or both inner and outer foreskin (p<0.05). In 2 donor samples, it was thinner than the inner foreskin (p<0.05). In 5 donor samples, the mean inner foreskin keratin thickness was thicker than that of the outer foreskin (p<0.05). Spearman rank analysis showed high correlation between mean keratin thicknesses of inner and outer foreskin for the same donor (black circles, figure 1C). This was not seen between the frenar band and the inner foreskin (gray boxes, figure 2B) or outer foreskin (data not shown).

Overall, there was no statistically significant difference in the level of keratin thickness between the inner (mean 14.67± SD 7.48 µm), outer (13.30± SD 8.49 µm), or frenar band (16.91± SD 12.42 µm) of the male foreskin (figure 2). The most heterogenous region overall was the frenar band. The largest difference in mean keratin thicknesses was between the frenar band and outer foreskin (3.61 µm). (Of note, donor #13 showed strong influence in all model diagnostics. However, the analysis upon removing this donor’s data showed similar results and thus is not presented.).

Concurrent, independent analysis at the Karolinska Institutet laboratory confirmed the findings reported above. For this analysis eleven of the 19 donor samples were randomly selected and each of the three foreskin sites were stained, imaged and analyzed in a blinded fashion (figure 3A). Readings from the two laboratories showed significant correlation (figure 3B, *r* = 0.444, 95% CI 0.1083–0.689, *p*<0.05). Donor sample #13 demonstrated a very thick keratin layer for both groups in the frenar band region. To exclude the possibility that these outliers skewed the analysis, we also calculated a Spearman rank correlation without this sample (figure 3B, shorter line). The two group readings remain significantly correlated in this secondary analysis.

## Discussion

We present here the first published analysis of foreskin keratinization in African men. Other studies using foreskin tissues collected in Rakai during a randomized, clinical trial of male circumcision for HIV prevention have shown correlations between foreskin surface area and HIV acquisition rates, as well as higher levels of anaerobic bacteria within the uncircumcised penile prepuce [3], [20], [21]. We found no differences in the thickness of keratin between the inner and outer foreskin surfaces or the frenar band among healthy HIV-uninfected men who underwent elective male circumcision as a prophylactic measure against HIV/STI acquisition. Two laboratories, one in the USA and the other in Sweden, concurrently imaged and analyzed the tissues in a blinded fashion. Measurements from both labs were highly correlated with one another. Differences between each laboratory’s mean measurements were likely due to intra-individual heterogeneity in foreskin keratin thickness (i.e., surveyed areas varied between the two groups).

These findings were consistent with our previous study using foreskin samples from donors in Chicago, IL, USA [15]. In that study, the mean keratin thickness of the inner and outer foreskin across all donors was 23.24 and 19.51 µm, respectively. The ratio of inner to outer keratin thickness was therefore 1.2∶1, similar to the 1.1∶1 ratio in this study. A major difference between these studies was the racial and ethnic background of donors: the Chicago cohort had a mixed ratio of Caucasian, Hispanic, and African-American donors, while the Rakai community is homogenously African. A few dermatologic studies have reported racial differences in skin keratin, but results have been inconsistent [22]. The results from our two studies cannot be directly compared as different methodologies were employed, but the absence of significant differences between inner and outer foreskin keratinization in both study populations suggests that race does not play a key role with respect to this characteristic.

We observed significant inter-individual differences in keratin thickness. These differences may have several causes, including genetic variation in epidermal keratinization and environmental exposures prior to circumcision. Although donors were screened for pre-existing STIs and evidence of inflammation, asymptomatic HPV and HSV infection is common in men from the Rakai community [4]. Importantly, there was significant intra-individual variation in keratin thicknesses throughout the foreskin. Keratin layers can change as the epithelial cells differentiate and regulate structural protein expression levels. This regulation is likely dependent on physical and environment factors. In addition, keratin can be physically sloughed off under mechanical stress (e.g., sexual intercourse). These small variations within a donor sample is an often over-looked phenomenon, and may bias studies based on limited observations or available tissue.

In this study, we improved our previously described methods for quantifying keratin thickness. In addition to highlighting filaggrin in the stratum corneum, we fluorescently labeled involucrin in the stratum granulosum. Filaggrin (filament aggregating protein) binds to keratin and is abundant in cornified layers of stratified squamous epithelia [23]. Involucrin is a small protein expressed by terminally differentiated epithelial cells and acts as a scaffold to which other structural proteins bind [24]. This molecular-based method provided us with an accurate visualization of the keratin layer using epifluorescent microscopy. As mentioned, we occasionally encountered areas where involucrin appeared superficial to filaggrin. This was most likely a staining artifact, but our study aim was to measure keratin thickness not quantify protein expression levels. Regardless, studies with transgenic mice over-expressing involucrin do not show any defects in epidermal barrier function and the clear presence of filaggrin in these areas support a robust keratin layer [25]. Finally, an IDL-based computer algorithm allowed for faster and more detailed measurements along the entire length of the imaged keratin layer.

Given our findings, other factors that distinguish the environment created by the existence of the foreskin need to be examined. For example, target cells are abundant in foreskin tissue and their responsiveness to chemokines and cytokines appear to be distinct in each region (i.e., cells in the inner foreskin appear to be more responsive than those in the outer foreskin) [10], [26], [27]. It is also possible that the scientific rationale for male circumcision does not lie in the foreskin itself and studies comparing circumcised to uncircumcised penile epithelia may yield more answers. Finally, while the sample size for our study was relatively small (19 participants), there was no trend to decreased keratin thickness in the inner foreskin and our results confirm other published analyses of foreskin keratin thicknesses [14], [15].

In summary, this multi-site, quantitative analysis of the foreskin keratin thickness in healthy Ugandan men showed no significant differences between keratin thickness of the inner, outer, and frenar band of the foreskin. Therefore, variation in keratin thickness alone cannot explain any putative differences in HIV acquisition across different regions of the foreskin.

## Supporting Information

Figure S1 **Keratin thickness measurements using IDL.** Epithelial foreskin tissue sections were labeled with fluorescently labeled α-filaggrin (red) and α-involucrin (green) antibodies. Cell nuclei labeled with DAPI (blue). Quick projected images obtained for analysis. (A) A user-drawn line provided the basal edge of the stratum. A similar line was drawn for the apical edge of the epithelium. (B) IDL-based program calculated distances between both apical-to-basal and basal-to-apical edges of keratin.
(TIF)
