# Supplementary figures and images for: No Difference in Keratin Thickness between Inner and Outer Foreskins from Elective Male Circumcisions in Rakai, Uganda
Source: PLoS One. 2012 Jul 18;7(7):e41271. doi: 10.1371/journal.pone.0041271 (PMC3399815; doi:10.1371/journal.pone.0041271)

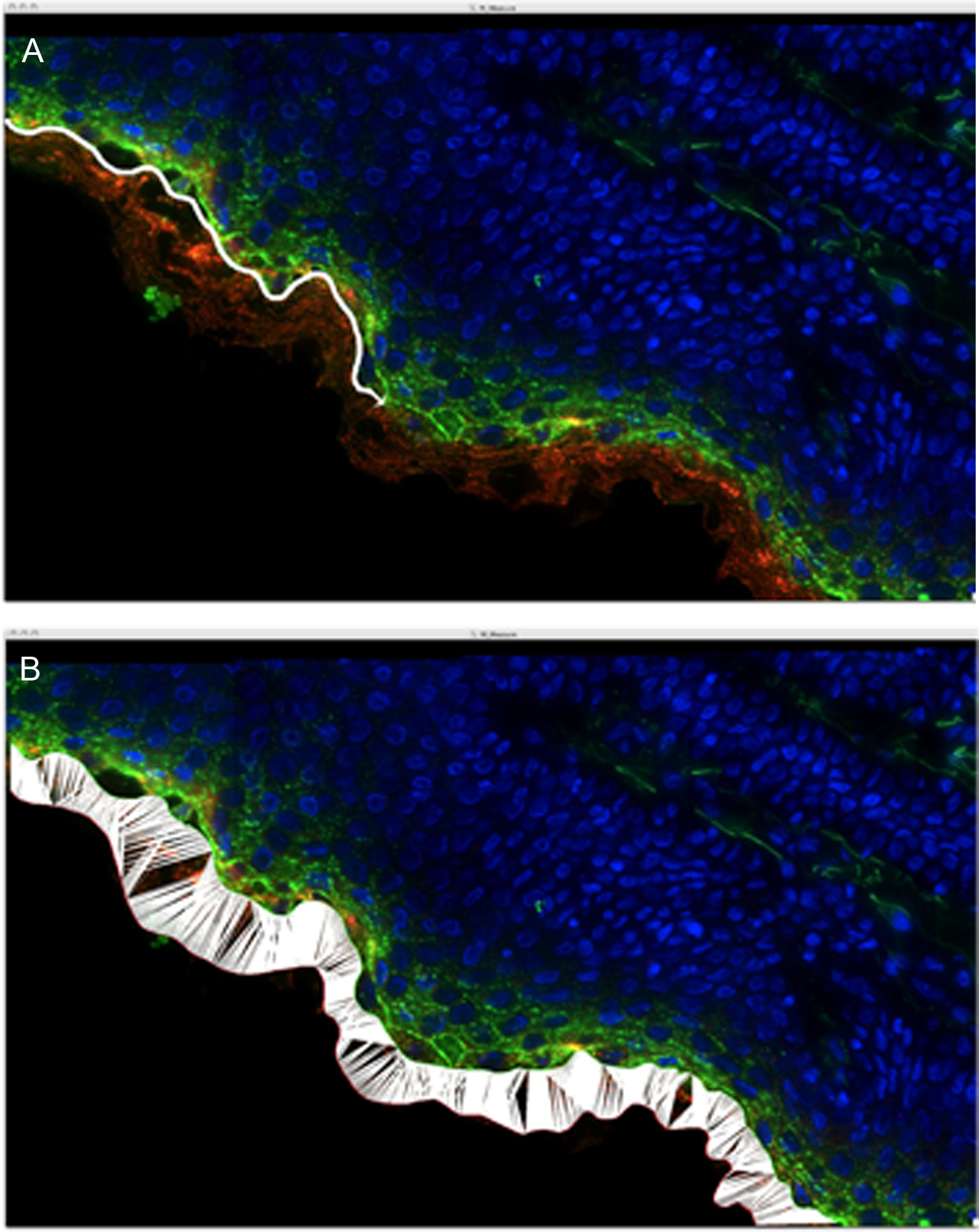

Supplement: Figure S1 — Keratin thickness measurements using IDL. Epithelial foreskin tissue sections were labeled with fluorescently labeled α-filaggrin (red) and α-involucrin (green) antibodies. Cell nuclei labeled with DAPI (blue). Quick projected images obtained for analysis. (A) A user-drawn line provided the basal edge of the stratum. A similar line was drawn for the apical edge of the epithelium. (B) IDL-based program calculated distances between both apical-to-basal and basal-to-apical edges of keratin. (TIF) [file pone.0041271.s001.tif]
